# Antitumor activity of melinjo (*Gnetum gnemon* L.) seed extract in human and murine tumor models in vitro and in a colon-26 tumor-bearing mouse model in vivo

**DOI:** 10.1002/cam4.520

**Published:** 2015-09-26

**Authors:** Narayanan K Narayanan, Kazuhiro Kunimasa, Yukio Yamori, Mari Mori, Hideki Mori, Kazuki Nakamura, George Miller, Upender Manne, Amit K Tiwari, Bhagavathi Narayanan

**Affiliations:** 1 Department of Environmental Medicine, New York University School of MedicineNew York, New York; 2 Cancer Chemotherapy Center, Japanese Foundation for Cancer ResearchKoto-ku, Tokyo, Japan; 3 Institution for World Health Development, Mukogawa Women’s UniversityNishinomiya, Hyogo, Japan; 4 School of Pharmacy and Pharmaceutical Sciences, Mukogawa Women’s UniversityNishinomiya, Hyogo, Japan; 5 Departments of Surgery and Cell Biology, New York University School of MedicineNew York, New York; 6 Department of Pathology, University of Alabama at BirminghamBirmingham, Alabama; 7 Department of Pharmacology and Experimental Therapeutics, College of Pharmacy & Pharmaceutical Sciences, University of ToledoToledo, Ohio

**Keywords:** Cancer prevention, gnetin C, in vitro and in vivo tumor models, melinjo seed extract, *trans*-resveratrol

## Abstract

Melinjo (*Gnetum gnemon* L.) seed extract (MSE) and its active ingredient gnetin C (GC), a resveratrol dimer, have been shown to possess a broad spectrum of pharmacological activities. In this study, we investigated the antitumor activity of MSE and GC using human and murine tumor cell culture models in vitro. The antitumor activity of GC was compared with *trans*-resveratrol (*t*RV), a stilbenoid polyphenol. Our results show that MSE and GC at clinically achievable concentrations significantly inhibited the proliferation of pancreatic, prostate, breast, and colon cancer cell types (*P* < 0.05), without affecting normal cells. Interestingly, GC exerts enhanced antitumor activity than that of *t*RV (*P* < 0.05). MSE and GC significantly induced apoptosis in all the cancer cells, indicating MSE and GC inhibit tumor cell growth by inducing apoptosis (*P* < 0.001). Our findings provide evidence that MSE might induce apoptosis in cancer cells via caspase-3/7-dependent and -independent mechanisms. However, GC might trigger both early and late stage apoptosis in cancer cells, at least in part by activating caspase 3/7-dependent mechanisms. Furthermore, the antitumor efficacy of MSE observed in vitro was also validated in a widely used colon-26 tumor-bearing mouse model. Oral administration of MSE at 50 and 100 mg/kg per day significantly inhibited tumor growth, intratumoral angiogenesis, and liver metastases in BALB/c mice bearing colon-26 tumors (*P* < 0.05). In conclusion, our findings provide evidence that MSE and GC have potent antitumor activity. Most importantly, we provide the first evidence that MSE inhibits tumor growth, intratumoral angiogenesis, and liver metastasis in a colon-26 tumor-bearing mice.

## Introduction

**Figure 1:**
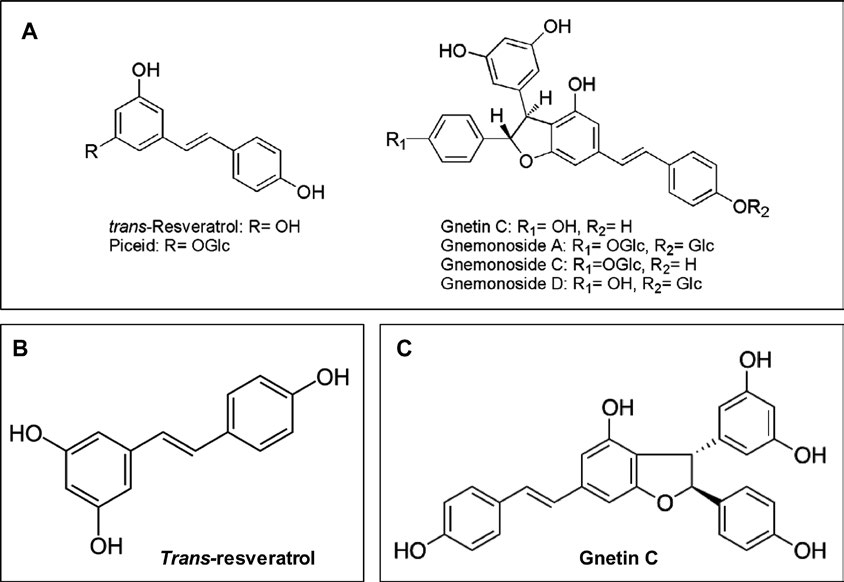
Chemical structures. (A) Stilbenoids from melinjo seeds. (B) *Trans*-resveratrol. (C) Gnetin C.

Melinjo (*Gnetum gnemon* L.), a member of the Gnetaceae family, is an arboreal dioecious plant widely cultivated in Southeast Asia 1. Its fruits, seeds, leaves, and flowers are edible 2,3. Melinjo seeds have a high nutritive value, and are consumed as a major food item by the Indonesian population. The melinjo seeds are rich in dimeric stilbenoids, such as gnetin C (GC) and its glucosides (gnemonosides A, C, and D), with *trans*-piceid glucoside as a minor constituent, including negligible amount (0.1%) of *trans*-resveratrol (*t*RV) 3–5 (Fig.1A). Melinjo seed extract (MSE) is sold as a nutritional supplement in Japan, and is now being introduced into the United States 6.

Recent studies indicate that the oral administration of MSE is well tolerated and efficiently absorbed, and does not produce significant adverse effects to in laboratory animals or humans. Tatefuji et al. 3 reported that repeated oral administration of MSE (1000 mg/kg per day) in rats had no observed adverse effect level (NOAEL). In addition, a rat bone marrow micronucleus test, which evaluates genotoxicity, was negative for MSE at doses up to 4000 mg/kg per day 3. Furthermore, a human study conducted in healthy volunteers reported that oral administration of MSE for 28 days, up to an oral dose of 5000 mg, daily, was well tolerated with no observable adverse events 7.

MSE has been reported to have a broad spectrum of pharmacological effects. For example, an ethanol extract of MSE seeds has antioxidant and radical scavenging activity, similar to that of ascorbic acid and dl-alpha-tocopherol 4,7. In addition, MSE inhibits the enzymes lipase and alpha-amylase and has antimicrobial activity against food microorganisms and enterobacteria 4. In mice, MSE produced an antimetabolic syndrome effect by suppressing a gain in body weight and improving insulin resistance 8. MSE and its active constituents also stimulated the immune response 9, inhibited angiogenesis 2, and prevented endothelial senescence 10. A recent double-blind randomized controlled clinical trial involving healthy adult males demonstrated that administration of 750 mg MSE for 8 weeks reduced serum uric acid levels at 4 and 8 weeks relative to levels in a group dosed with a placebo 5. It has been reported that MSE may decrease serum uric acid regardless of insulin resistance, and may improve lipid metabolism by increasing levels of HDL cholesterol 5.

Gnetin C, a dimeric stilbenoid abundant in MSE (Fig.1B), is reported to have advantages over *t*RV (3,5,4′-trihydoxy-*trans*-stilbene), a stilbenoid polyphenol in red wine (grape skins) 11. It has been reported that GC may be the most effective component among the MSE derivatives, since it suppressed the angiogenesis-related endothelial cell functions and tumor-induced angiogenesis significantly compared to its glycosides (gnemonosides A, C, and D) 2. Considering the relative amounts of GC, 28.0 mg/g (61.7 *μ*mol/g), GL, a minor component (4.95 *μ*mol/g), and *t*RV (5.26 *μ*mol/g) in MSE 7, the antiangiogenic effects of MSE have been attributed to GC 2. While the literature on the effects of *t*RV on cancer cells is abundant, numerous studies indicate that it has poor bioavailability 12–15. Moreover, the rapid metabolism of *t*RV is an obstacle to using it in human preclinical studies 16. In contrast, GC has excellent bioavailability. For example, the concentration of GC was about sixfold higher than that of *t*RV in streptozotocin-treated and control mice after administration of an ethanol extract of *G. gnemon* seeds 10. In addition, the oral administration of 1000 mg of MSE powder to healthy volunteers indicated that GC was maintained in plasma longer than 96 h, and had a mean residence time (MRT) of 36 h, relative to 24 h for *t*RV, with a *t*_max_ of 12 h and an MRT of 14 h 7. GC inhibits tyrosinase activity, and thus suppresses melanin biosynthesis, in B16 cells 17. Given the broad spectrum of pharmacological effects reported for MSE and GC, we postulate that MSE and GC have properties that might prevent and/or treat cancer.

Cancer remains the second most common cause of death in the United States, accounting for nearly one of every four deaths 18. There is an increase in the cancer burden worldwide 19. Despite decreases in the incidence and mortality rates for most types of cancer (including lung, colorectal, prostate, and female breast cancer) in the United States and many other Western countries, the number of cancer cases and deaths is projected to more than double worldwide over the next 20–40 years 20,21. Notwithstanding the steady progress in drug discovery and effective prevention, the treatment of most cancers remains challenging. The use of several chemotherapeutic drugs, including nontargeted and molecularly targeted agents currently employed in cancer therapy, is severely limited by their adverse side effects 22. On the other hand, natural compounds that selectively exert cytotoxic effect on cancer cells without affecting normal cells have attracted considerable attention as cancer chemopreventive and cancer therapeutic agents 23–25. Various dietary components found in plant-derived foods have been recognized for their anticarcinogenic properties 26–28. While various plant-derived molecules have shown anticancer properties, only a few with anticancer potential are currently undergoing clinical trials 29,30. Therefore, there is a need to identify plant-derived, bioactive food components with anticancer potential to prevent and/or treat human cancers.

In the present work, we investigated the anticancer potentials of both MSE and its putative active component GC by using various human and murine tumor cell types derived from the pancreas, colon, breast, and prostate for in vitro analysis. Nonmalignant cells were used to determine whether MSE or GC selectively targets cancer cells without affecting normal cells. Since GC is a dimeric stilbenoid (resveratrol dimer derivative), *t*RV was used as a control in the in vitro experiments. Apoptosis assay was performed to determine whether MSE or GC inhibits tumor cell growth by inducing apoptosis. We also performed luminescent caspase-3/7 and caspase-9 assays, to understand whether MSE or GC induces programmed cell death (apoptosis) by activating the caspases. Furthermore, the antitumor efficacy of MSE observed in vitro was also validated in a widely used colon-26 tumor-bearing mouse model. Here, we report that MSE and GC have anticancer potential, and might be useful to prevent and treat certain cancers. Most importantly, we also provide evidence that MSE inhibits tumor growth, angiogenesis, and liver metastasis in a colon-26 tumor-bearing mice model.

## Materials and Methods

### Chemical agents

MSE and GC were provided by the Yamada Bee Company, Japan, under MTA agreements with Drs. Kunimasa and Narayanan at the Japanese Foundation for Cancer Research and New York University School of Medicine, respectively. *t*RV was purchased from Sigma-Aldrich (St. Louis, MO). The MSE used in this study was prepared from MSE powder (Lot. YMP-M-100710) containing GC (3.68%) and its glucosides (gnemonoside A, 14.00%; gnemonoside D, 5.44%), and RES (0.11%). MSE powder was dissolved in Dimethyl sulfoxide (DMSO) at up to 100 mg/mL (stock solution). The solution was sonicated briefly prior to use. Stock solutions of GC and *t*RV were dissolved in DMSO at up to 50 mmol/L. Stock solutions were stored at –20°C. Subsequently, various dilutions of MSE, GC, or *t*RV were prepared fresh by diluting each stock solution in the cell culture medium.

### In vitro antitumor activity assessment

#### Cell types

Cell types remain the main tools for screening procedures that could provide information necessary to select agents with potentially useful anticancer properties for further chemical and pharmacological investigations. Therefore, in the present investigation, a panel of selected human and murine cancer cell types was used to evaluate the antitumor activity of MSE or GC. Human prostate (LNCaP and PC-3), breast (MCF-7), and colon (HT-29) cancer cell types used in our experiments were obtained from the American Type Culture Collection (ATCC, Manassas, VA) and were authenticated by ATCC. Human (PANC-1 and AsPC-1) and mouse (Pan-02) pancreatic cancer cells were provided and authenticated by Dr. George Miller (New York University Medical Center). Murine PTEN-CaP8 prostate cancer cells, derived from a PTEN-KO mouse adenocarcinoma 31, were provided and authenticated by Dr. Hong Wu (University of California, Los Angeles, CA). The BALB/c-mouse-derived colon adenocarcinoma cell line (colon-26) was provided and authenticated by the RIKEN BRC through the National Bio-Resource Project of the MEXT, Japan. In addition, we examined the effect of MSE or GC on the nonmalignant cell types, HEK-293T (human embryonic kidney cells that exhibit epithelial morphology), and RWPE-1 (normal human prostate epithelial cells). HEK-293T and RWPE-1 cells were provided and authenticated by Drs. Wei Dai and Susan Logan, respectively, at the New York University Medical Center.

#### Cell culture

Human PANC-1 and AsPC-1 cells were cultured in Dulbecco’s modified Eagle’s medium (DMEM) with 3.7 mg/mL sodium bicarbonate, 10% fetal bovine serum (FBS, Gibco, Invitrogen, Carlsbad, CA), 100 units/mL penicillin, and 100 *μ*g/mL streptomycin. Human LNCaP, PC-3, MCF-7, and HT-29 cells and mouse Pan-02 and colon-26 cells were grown in RPMI (Invitrogen) with 10% FBS, 100 units/mL of penicillin, and 100 *μ*g/mL of streptomycin or kanamycin. PTEN-Cap8 cells were grown and maintained in DMEM supplemented with 10% FBS, 25 *μ*g/mL of bovine pituitary extract, 5 *μ*g/mL of bovine insulin, and 6 ng/mL of recombinant human epidermal growth factor. All cell types were maintained at 37°C in a humidified 5% carbon dioxide and 95% air incubator. All cells were passaged approximately two times/week. Cells at 80–90% confluency were used for treatments 32.

#### MTS cell proliferation assay

The CellTiter 96® AQueous One Solution cell proliferation assay [3-(4,5-dimethylthiazol-2-yl)-5-(3-carboxymethoxyphenyl)-2-(4-sulfophenyl)-2H-tetrazolium, inner salt; MTS] was performed to investigate the anticancer potential of MSE and its active component GC. Briefly, human PANC-1, AsPC-1, HT-29, MCF-7, LNCaP, Du145, and PC-3 cells, and mouse Pan-02, colon-26, and PTEN-CaP8 cells were plated in 96-well plates (3 × 10^3^ cells and 200 *μ*L of fresh complete medium per well) for 24 h before treatment. Fresh complete medium (200 *μ*L) containing various concentrations of MSE (0, 1.25, 12.5, 25, 50, 100, 200, and 400 *μ*g/mL) were added into each well, and the cells were treated for 24, 48, and 72 h. MSE contains 61.7 *μ*mol/g of GC 7; therefore, we selected 0–100 *μ*mol/L GC to represent the GC concentration present in a typical MSE. Since GC is a dimeric stilbenoid (resveratrol dimer derivative), *t*RV was used as a control to compare the antitumor activity of GC. For GC or *t*RV treatment, cells were plated in 96-well plates, at 3 × 10^3^ cells per well, and exposed to various concentrations of GC or *t*RV (0, 1, 3, 6, 12, 25, 50, and 100 *μ*mol/L) for 24, 48, and 72 h. Furthermore, nonmalignant HEK-293T and RWPE-1 were also treated with various concentrations of MSE, GC, or *t*RV, as outlined above. The Cell Titer 96 AQ_ueous_ Non-Radioactive Cell Proliferation Assay Kit (Promega, Madison, WI) was used according to the manufacturer’s protocol to determine the number of viable cells after treatment with MSE, GC, or *t*RV. Absorbance was read at 490 nm with an ELISA microplate reader, the SpectraMax M2 dual-monochromator (Molecular Devices, Sunnyvale, CA). For each treatment group, triplicate wells were analyzed to assess cell viability. The percentage of viable cells was determined using the following formula: (Absorbance test well/Absorbance control well) × 100. All experiments were performed in triplicate.

#### DAPI staining assay for apoptosis detection

An apoptosis assay was performed to investigate whether MSE or GC inhibit tumor cell growth by inducing apoptosis. The rate of apoptosis induced by MSE or GC was determined using DAPI (4′,6-diamidino-2-phenylindole, Sigma-Aldrich) staining. Briefly, a panel of human cancer cell types derived from the pancreas (PANC-1), colon (HT-29), breast (MCF-7), and prostate (PC-3), as well as nonmalignant cells (HEK-293T and RWPE-1) grown in 35-mm dishes cells were treated for 24–48 h with IC_50_ concentrations of MSE or GC (determined by dose–response experiments in this study). In a parallel experiment, cells were treated with IC_50_ concentrations of *t*RV that served as a control to compare the apoptotic activity of GC. After treatment, floating and adherent cells were fixed in 10% formalin for 15 min. After a washing with phosphate-buffered saline (PBS), cells were treated with 0.1% Triton X-100, 4 mol/L HCl, and sodium tetraborate; each treatment was performed for 15 min and was followed by a PBS wash. Cells were then stained with DAPI in 80% methanol for 30 min and again washed with PBS. The cells were viewed under a fluorescence microscope (AX70-Olympus) with ×40 magnification.

DAPI-positive cells with characteristic nuclear condensation and DNA strand breaks for apoptosis were counted from 10 identical fields. The percentage of apoptosis in the MSE and GC treatment groups were normalized relative to the vehicle (DMSO) treatment. The percentages of apoptotic cells were determined from three identical experiments and compared with controls.

#### Caspase-3/7 and caspase-9 assays

To explore the possible mechanisms by which MSE or GC may regulate apoptosis in cancer cells, specifically, to determine whether MSE or GC induces apoptosis, by activating the caspase family of cysteine proteases, we performed luminescent caspase-3/7 and caspase-9 assays in a 96-well format (Promega). HT-29 and PC-3 cells were plated at 2 × 10^3^ cells/well for HT-29 cells, and at 4 × 10^3^ cells/well for PC-3 cells. After a 24-h preincubation, the cells were treated with vehicle (DMSO), MSE (25 and 50 *μ*g/mL), or GC (6.13 and 12.5 *μ*mol/L) around the IC_50_ concentration of each cell line (determined by a dose–response study for 48 h) for 6, 24, and 48 h. In addition, to determine the effect of MSE or GC on caspase-3/7 and -9 over a long period of time, cells were also treated for 72 h. The caspase activities were shown as % of control at each time point. All experiments were performed in duplicate for the caspase-Glo 9 assay or in triplicate for the caspase-Glo 3/7 assay.

### In vivo antitumor efficacy assessment

#### Animals

Female BALB/c mice (8 weeks of age) were purchased from Japan SLC, Inc., Japan. The mice were maintained in a pathogen-free environment and all experiments in this study were carried out in accordance with the protocol approved by the Institutional Animal Care and Use Committee of Mukogawa Women’s University, Hyogo, Japan.

#### Efficacy evaluation of MSE in colon tumor model

The antitumor efficacy of MSE was assessed in a murine colon carcinoma model in vivo. Briefly, colon-26 cells (1 × 10^6^) in 50 *μ*L of PBS were implanted subcutaneously into the right back of female BALB/c mice on day 1. Before tumor cell engraftment, subconfluent colon-26 cells were harvested and resuspended in PBS to a density of 1 × 10^6^ cells/mL. Prior to cell inoculation, cell viability was examined using a 0.4% trypan blue exclusion assay (viable cells >90%). Vehicle (corn oil) or MSE (50 or 100 mg/kg per day) was orally administered to control mice (*n* = 6) or MSE mice (*n* = 7 each), respectively, from day 2 to 25. All mice were monitored daily for their general health status and weighed. Tumor size was measured every 4 days from day 10. Tumor volume (mm^3^) was calculated by the following formula: (short diameter)^2^ × (large diameter) × 0.52. On day 26, all mice were euthanized; subcutaneous tumor tissues were then extirpated, weighed, and fixed in 10% neutral-buffered formalin for histopathological and immunohistochemical (IHC) evaluations. Formalin-fixed, paraffin-embedded tissues were also sectioned (4 *μ*m) and stained with CD31 to evaluate the effect of MSE on intratumoral microvessels. The effect of MSE on the tumor-induced intratumoral microvessel density (MVD) was assessed and expressed as CD31-positive area (%).

#### Efficacy evaluation of MSE against liver metastasis in intrasplenic colon tumor model

To evaluate the effect of MSE on metastasis, colon-26 tumor cells were implanted into the spleen of female BALB/c mice to produce liver metastasis (tumor nodules). Briefly, colon-26 cells (1 × 10^6^ cells/100 *μ*L of PBS) were implanted into the spleens of female BALB/c mice on day 1. Vehicle (corn oil) or MSE (50 or 100 mg/kg per day) were orally administered (*n* = 7 or 8 mice per group) from day 2 to 20. Female BALB/c mice injected with PBS served as negative control (*n* = 7). On day 21, mice were euthanized, and liver tissues were extirpated, weighed, and fixed with 10% neutral-buffered formalin. The formalin-fixed, paraffin-embedded tissues were sectioned (4 *μ*m) and stained with H&E to evaluate the effect of MSE on intraliver micrometastases. The metastatic index was estimated by calculating the mean of metastatic grades on the liver surface in each group. Metastatic grade on the liver surface was classified from grade 0 to 3: no metastatic nodule (grade 0), one to three metastatic nodules (grade 1), four to six metastatic nodules (grade 2), and seven or more metastatic nodules (grade 3). Metastatic grade in the liver cross-section was classified from grade 0 to 3: no metastatic focus (grade 0), one to five metastatic foci (grade 1), six to ten metastatic foci (grade 2), and eleven or more metastatic foci (grade 3).

### Statistical analysis

The data presented for all in vitro studies were representative of three sets of experiments. All the data were presented as mean ± SE, unless otherwise indicated. The effects of MSE, GC, or *t*Rv on tumor cell proliferation, apoptosis detection, and caspase activities were compared using analysis of variance (ANOVA) and Student’s *t*-test for pairwise comparisons. For the in vivo assessment of body weight, tumor volume/weight, tumor burden, and assessment metastasis index on the liver surface and in the liver, Dunnett’s multiple comparison test was used after ANOVA to analyze differences between the MSE-treated and control groups 33. For all analyses, a *P* < 0.05 was considered to be statistically significant. All data analyses were performed with GraphPad Prism 5 statistical software (San Diego, CA).

## Results

### MSE and GC inhibit the proliferation of human and murine tumor cells

**Figure 2:**
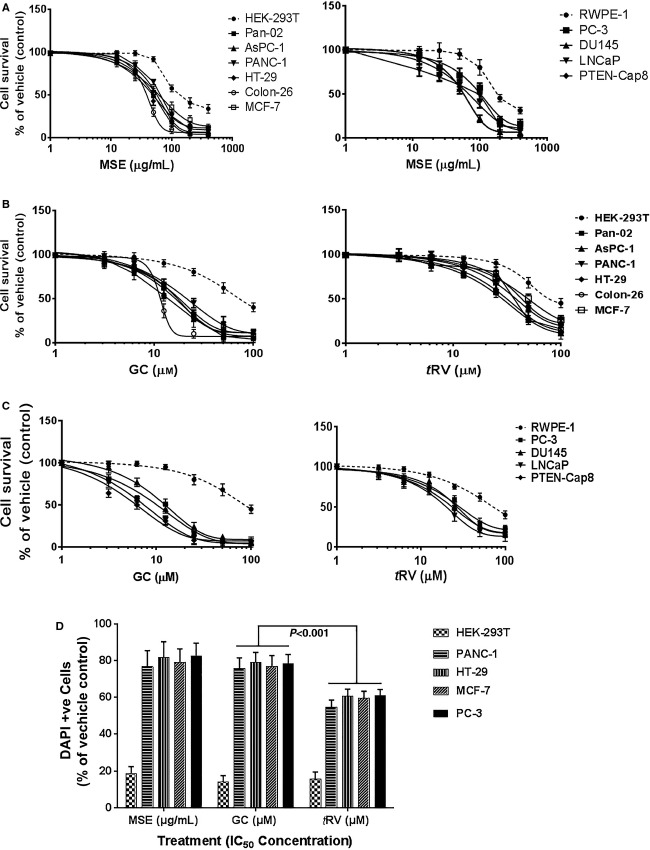
Effect of MSE or GC on cancer cell proliferation and apoptosis. (a-c) MSE and GC inhibited proliferation of all human and mouse cancer cells in a concentration -dependent manner. GC significantly inhibited cancer cell proliferation compared with *t*RV. MSE or GC did not adversely affect normal cells. Data presented are means ± SD, and are representative of three independent experiments. (d) The bar graph shows the rate of apoptosis (percentage of apoptotic cells determined by DAPI staining) in the MSE and GC treatment groups, normalized to the vehicle (DMSO) treatment. DAPI-positive cells with characteristic nuclear condensation and DNA strand breaks for apoptosis were counted from 10 identical fields using a fluorescence microscope (Olympus) with × 40 magnifications. In contrast to the profound apoptosis induction in cancer cells, only marginal or very low levels of apoptosis were detected after normal cells were incubated with MSE or GC. A significant increase in apoptosis induction was observed after incubation with GC compared to *t*RV, p<0.001. The data are presented as the mean ± SD and are representative of three independent experiments.

The anticancer potential of MSE and GC was investigated with a panel of human and mouse cancer cell types, as described in the Materials and Methods section. The anticancer potential of GC, a dimeric stilbenoid (a resveratrol derivative) was compared with *t*RV. As shown in Figure2A–C, MSE and GC inhibited the growth of human and mouse cancer cell types in a dose-dependent manner. The IC_50_ values are summarized in Table1. MSE and GC showed antitumor efficacy against various human and murine cancer cell types. However, colon (HT-29 and colon-26), breast (MCF-7), and prostate (LNCaP, DU145, and PC-3) cancer cells were highly sensitive to MSE with the IC_50_ values of 39.37 ± 4.9 *μ*g/mL (HT-29), 36.3 ± 4.9 *μ*g/mL (colon-26), 37.3 ± 0.9 *μ*g/mL (MCF-7), 34.26 ± 0.11 *μ*g/mL (LNCaP), 39.38 ± 3.62 *μ*g/mL (DU145), 38.26 ± 0.24 *μ*g/mL (PC-3), and 35.91 ± 0.13 *μ*g/mL (PTEN-CaP8) when compared with pancreatic cancer cells (Table1). GC was significantly more potent in inhibiting the growth of prostate cancer cells, with the IC_50_ values of 8.95 ± 0.92 *μ*mol/L (LNCaP), 9.85 ± 2.60 *μ*mol/L (DU145), 10.28 ± 0.79 *μ*mol/L (PC-3), and 9.01 ± 0.15 *μ*mol/L (PTEN-CaP8) compared with pancreas, colon, and breast cancer cells (Table1). GC significantly inhibited cell proliferation in human and murine cancer cells compared with *t*RV (*P* < 0.05), indicating that GC is more efficacious than *t*RV (Table1). The IC_50_ values of MSE or GC are significantly lower in cancer cells compared with those of normal epithelial cell, suggesting that MSE and GC exert less cytotoxic effect on normal epithelial cells and selectively kill cancer cells (Table1).

**Table 1:** IC_50_ values of melinjo seed extract (MSE), gnetin C (GC), and resveratrol against cancer cell proliferation

Cell lines	Description	MSE (*μ*g/mL) (mean ± SE)	GC (*μ*mol/L) (mean ± SE)	*t*RV (*μ*mol/L) (mean ± SE)
PANC-1	Human pancreatic cancer cells	61.27 ± 2.58	16.29 ± 1.11†	36.26 ± 0.66
AsPC-1	Human pancreatic cancer cells	53.74 ± 3.2	13.83 ± 0.92†	31.14 ± 1.38
Pan-02	Mouse pancreatic cancer cells (NCI)	48.42 ± 3.01	12.22 ± 1.45†	29.16 ± 0.84
PC-3	Human prostate cancer cells (AR negative, androgen independent)	38.26 ± 0.24*	10.28 ± 0.79*†	17.79 ± 1.32
DU-145	Human prostate cancer cells (AR negative, androgen independent)	39.38 ± 3.62*	9.85 ± 2.60^*^†	20.46 ± 4.92
LNCaP	Human prostate cancer cells (AR positive, androgen dependent)	34.26 ± 0.11*	8.95 ± 0.92*†	13.26 ± 0.88
PTEN-CaP8	Mouse prostate cancer cells derived from the adenocarcinoma of PTEN null mice	35.91 ± 0.13*	9.01 ± 0.15*†	14.82 ± 1.13
MCF-7	Human breast cancer cells	37.3 ± 0.9*	13.13 ± 0.61*†	31.34 ± 6.2
HT-29	Human colon cancer cells	39.33 ± 4.9*	11.78 ± 1.45*†	38.28 ± 0.55
Colon-26	Mouse colon cancer cells (RIKEN BRC, Japan)	36.3 ± 4.9*	11.3 ± 0.6^*^†	37.0 ± 0.6
HEK-293T	Human embryonic kidney epithelial cells	87.37 ± 2.34**	85.54 ± 1.23	91.31 ± 8.09
RWPE-1	Normal human prostate epithelial cells	89.70 ± 1.76**	87.89 ± 5.25**	96.17 ± 6.55

Cell survival assay: MTS assay was utilized to assess the tumor cell proliferation as described in the Materials and Methods section. The antitumor activity of GC was compared with *t*RV.

Statistical analysis: The data presented for all in vitro studies were representative of three sets of experiments. All the data were presented as mean ± SE, unless otherwise indicated. The effects of MSE, GC, or *t*Rv on tumor cell proliferation were compared using ANOVA and Student’s *t*-test for pairwise comparisons. For all analyses, a *P*-value <0.05 was considered to be statistically significant. All data analyses were performed with GraphPad Prism 5 statistical software (San Diego, CA).

MSE significantly inhibits cell proliferation in cancer cells compare to controls. More specifically, colon, breast, and prostate cancer cell were highly sensitive to MSE when compared to breast and pancreatic cancer cell

* *P* < 0.001. GC, an active component of MSE, significantly inhibited cell proliferation in cancer cells compared to *t*RV.

*† *P* < 0.05.

** IC_50_ values of MSE or GC are significantly higher in normal epithelial cells compared to that in cancer cells.

### MSE and GC inhibit tumor cell growth by inducing apoptosis

To demonstrate MSE or GC-mediated apoptosis induction, apoptosis was assessed using a panel of human cancer cell types, derived from the pancreas (PANC-1), colon (HT-29), breast (MCF-7), and prostate (PC-3), including nonmalignant cells (HEK-29T) treated with or without the IC_50_ concentrations of MSE or GC (Table1). DAPI staining, a reliable apoptotic assay 34, was used to measure the apoptotic cells induced by MSE or GC. The cells treated with *t*RV were used to compare the apoptotic activity of GC. Increases in the DAPI uptake and apoptotic cells with specific morphological and nuclear material changes characteristic of apoptotic cells were quantified (Fig.2D). Results from DAPI staining show that MSE and GC significantly induced apoptosis in all cancer cells (*P* < 0.001), indicating that MSE and GC inhibit tumor cell growth by inducing apoptosis. GC caused a significantly higher level of apoptosis than *t*RV (Fig.2D), *P* < 0.05). In contrast to the profound apoptosis produced in cancer cells, only marginal or very low levels of apoptosis were detected in normal cells treated with MSE or GC.

### MSE and GC selectively activate caspases

In an effort to better understand the possible mechanisms by which MSE or GC may regulate programmed cell death or apoptosis in cancer cells, we assessed the activities of caspases, a family of cysteine–aspartic proteases, which play a central role in apoptosis 35,36. We performed luminescent caspase-Glo 3/7 and caspase-Glo 9 assays to measure caspase-3/7 and -9 activities that are associated with the extrinsic and intrinsic apoptotic pathways, respectively. We measured time-dependent caspase-3/7 and -9 in human HT-29 and PC-3 cells treated with MSE or GC around their IC_50_ concentrations (Table1). We observed that MSE treatment (25 or 50 *μ*g/mL) had no significant effect on caspase 3/7 in HT-29 cells (Fig. S1A), suggesting that MSE may induce apoptosis via caspase-3/7-independent mechanisms in poorly metastatic colon cells (HT-29). However, incubation of 50 *μ*g/mL of MSE after a prolonged exposure (72 h) activated caspase-3/7 in metastatic prostate cancer cells (PC-3), *P* < 0.05 (Fig. S1B), indicating that MSE might induce late stage apoptosis in prostate cancer cells via extrinsic apoptotic pathway. GC significantly activated caspase-3/7 at 6 and 24 h in HT-29 cells, *P* < 0.05 (Fig. S1C), suggesting that GC might induce early stage apoptosis in HT-29 cells. Interestingly, GC significantly activated caspase-3/7 at the 24 and 72 h time points in PC-3 cells, *P* < 0.05 (Fig. S1D), suggesting that GC might induce both early and late stage apoptosis in PC-3 cells. Thus, our findings provide evidence that GC might trigger both early and late stage apoptosis in cancer cells via extrinsic apoptotic pathway, at least in part by activating caspase 3/7-dependent mechanisms. However, caspase-9 (intrinsic pathway), an upstream protease of caspase-3/7 was not activated by MSE or GC in either HT-29 or PC-3 cells (Fig. S2A–D), suggesting that a proteolytic cascade involving activation of all caspases may not be a common or essential feature of MSE or GC-induced apoptosis.

### MSE inhibits tumor growth in colon-26 tumor-bearing mice in vivo

**Figure 3:**
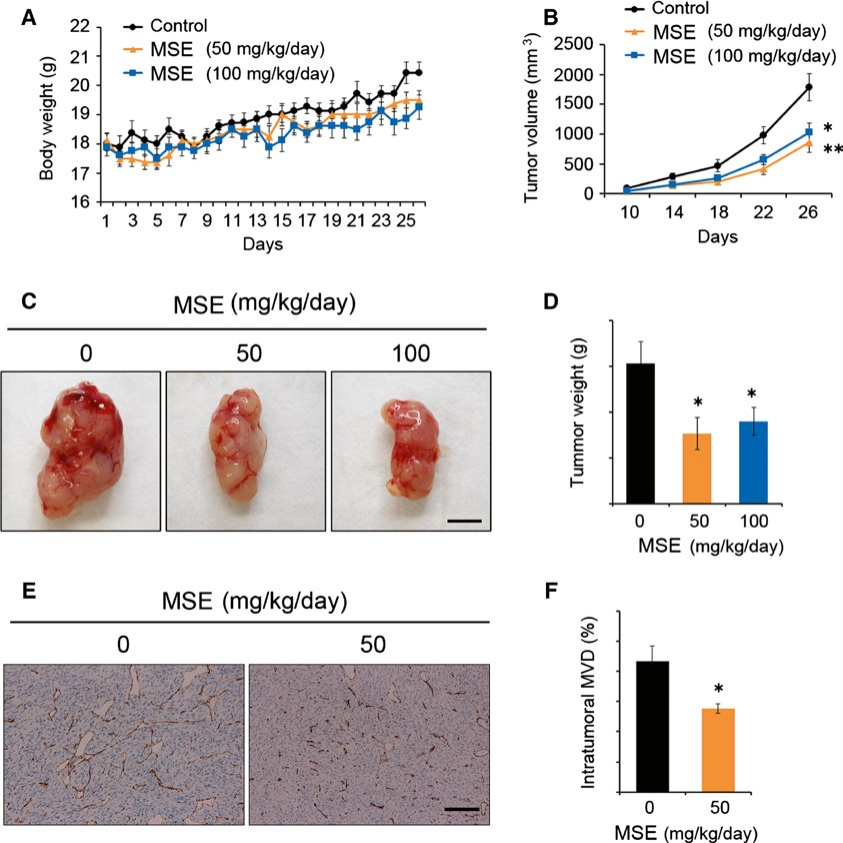
Effect of melinjo seed extract (MSE) on murine colon tumor growth in BALB/c mice. (A) The body weight gain between MSE treatment (50 or 100 mg/kg per day, *n* = 7 mice in each group) versus control (*n* = 6 mice) groups in BALB/c mice bearing colon-26 tumors. (B) The colon tumor volume at the termination of the experiment. MSE significantly decreased the colon tumor growth compared with control (*P < 0.05 and **P < 0.01). However, no significant difference was observed between the MSE doses. (C) Representative tumor images of control and MSE treatments. (D) Colon tumor weight at the termination of the experiment. The bar graph indicates a significant decrease in the tumor weight (*P < 0.05). (E) Representative IHC images of CD31 staining on the effect of MSE on the intratumoral microvessels (MVD). (F) The bar indicates the quantification of intratumoral MVD expressed as CD31-positive areas (%) (*P < 0.05). Data are shown as mean ± SE. Dunnett’s test subsequent to ANOVA was performed for (B) and (D), and Student’s *t*-test for (F).

To determine the in vivo efficacy of MSE, its antitumor activity was assessed in a murine colon carcinoma model. The oral administration of MSE at 50 and 100 mg/kg per day produced a slight weight gain in BALB/c mice (Fig.3A), with no signs of toxicity, behavioral abnormality, or animal death observed. The oral administration of MSE at 50 and 100 mg/kg per day significantly suppressed tumor volume and weight (*P* < 0.05; Fig.3B–D). However, there were no significant differences in the tumor volume and tumor weight between the 50 and 100 mg/kg per day doses. To clarify the contribution of the antiangiogenic property of MSE reported earlier 2 to its tumor-suppressive effect, we determined the intratumoral microvascular density (MVD) by quantifying the areas positively stained for CD31 (endothelial marker) (Fig.3E and F). MSE treatment at 50 mg/kg per day significantly decreased the MVD, relative to the control (vehicle-treated) group. These results indicate that MSE suppresses the growth of colon tumors, as well as inhibiting tumor-induced angiogenesis.

### MSE suppresses liver metastasis in colon-26 tumor-bearing mice in vivo

**Figure 4:**
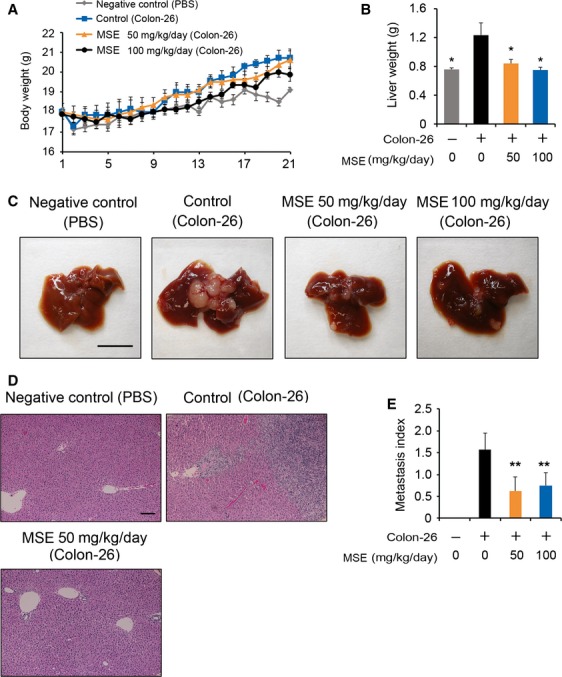
Effect of melinjo seed extract (MSE) on liver metastasis in BALB/c mice bearing murine colon-26 tumor. (A) Body weight gain in BALB/c mice of control and MSE treatment groups during the experimental period. (B) Changes in liver weights that were extirpated on day 21 (*P < 0.05). (C) Representative images of liver metastasis of intrasplenically injected colon-26 cells in the negative control, control, and MSE-treated mice. (D) Intraliver metastasis of colon-26 cells were confirmed by H&E staining. (E) The bar indicates the quantification of metastatic index. Metastatic index was estimated by calculating the mean of metastatic grades on the liver surface in each group. Data are shown as mean ± SE (*n *=* *7 for control groups and *n* = 8 for MSE treatment groups). **P < 0.01, compared with the control group (Dunnett’s test subsequent to ANOVA).

Liver metastases are frequently inoperable and significantly affect the prognoses of patients with cancer 37. Therefore, to determine the effect of MSE on liver metastasis, colon-26 tumor cells were implanted into the spleens of female BALB/c mice to produce liver metastases (tumor nodules). Similar to the colon-26 tumor model, the oral administration of MSE at 50 and 100 mg/kg per day to BALB/c mice produced a significant weight gain, although a small decrease in total weight gain was observed in the negative control group (injected with PBS only; Fig.4A). At the time of necropsy, however, mice in all groups showed no signs of toxicity. The liver weights of the mice intrasplenically transplanted with colon-26 cells and administered with MSE (50 and 100 mg/kg per day) were significantly lower to those of the control mice with colon-26 cells, *P* < 0.05 (Fig.4B). The treatment of mice with MSE (50 and 100 mg/kg per day) reduced both the number of metastatic nodules on the liver surface and the frequency of intraliver metastatic foci (Fig.4C–E). The grade classification on the liver surface and/or in the liver revealed a lower tendency of metastasis in MSE-treated mice, relative to the control group (Table2). These results indicate that MSE suppresses liver metastasis of colon cancer cells in vivo.

**Table 2:** Grade classification for metastasis on the liver surface and in the liver

	*n*	No. of each metastatic grade on the liver surface^1^				No. of each metastatic grade in the liver^2^			
		Grade 0	Grade 1	Grade 2	Grade 3	Grade 0	Grade 1	Grade 2	Grade 3
Negative control^3^	8	8	0	0	0	8	0	0	0
Control^4^	7	1	2	3	1	1	2	2	2
MSE (50 mg/kg per day)^5^	8	5	1	2	0	6	1	0	1
MSE (100 mg/kg per day)^5^	8	4	2	2	0	NT	NT	NT	NT

Assessment of metastasis: On day 21, mice were euthanized, and liver tissues were extirpated, weighed, and fixed with 10% neutral-buffered formalin. The formalin-fixed, paraffin-embedded tissues were sectioned (4 *μ*m) and stained with H&E to evaluate the effect of MSE on intraliver micrometastases. The metastatic index was estimated by calculating the mean of metastatic grades on the liver surface in each group.

Statistics: For the in vivo assessment of metastasis index on the liver surface and in the liver, Dunnett’s multiple comparison test was used after ANOVA to analyze differences between the MSE-treated and control groups. All data analyses were performed with GraphPad Prism 5 statistical software (San Diego, CA). *n *= number mice per group; NT, not tested. Statistical comparison of the distribution of metastatic grades of control versus each of the MSE treatments in liver and liver surface shows that there is significant inhibition of liver metastasis (*P* < 0.05). However, there is only a moderate inhibition in the distribution of grades in the treatment versus control on the liver or the liver surface metastasis. Future studies will be designed with larger sample size (*n*) to obtain firm conclusion on the inhibition of the metastatic grades.

1 Grade classification: grade 0, no metastatic nodule; grade 1, one to three metastatic nodules; grade 2, four to six metastatic nodules; grade 3, seven or more metastasis nodules.

2 Grade classification: grade 0, no metastatic focus; grade 1, one to five metastatic foci; grade 2, six to ten metastatic foci; grade 3, eleven or more metastatic foci.

3 Mice with PBS-only fed with rodent chow without MSE (melinjo seed extract).

4 Mice with colon-26 cells fed with rodent chow without MSE.

5 Mice with colon-26 cells fed with rodent chow mixed with MSE.

## Discussion

In the present study, we established the antitumor potentials of both MSE and GC in a panel of human and mouse cancer cell types derived from the pancreas, colon, breast, and prostate in vitro. GC may be the most effective component among the MSE derivatives, given that it suppressed the angiogenesis-related endothelial cell functions and tumor-induced angiogenesis significantly more effectively than its glycosides (gnemonosides A, C, and D) 2. Considering the relative amounts of GC, 28.0 mg/g (61.7 *μ*mol/g), GL, a minor component (4.95 *μ*mol/g), and *t*RV (5.26 *μ*mol/g) in MSE 7, the antiangiogenic effects of MSE have been attributed to GC 2. Hence, we investigated the anticancer potentials of both MSE and its putative active component GC using various human and murine tumor cell culture models. The *t*RV was used to compare the anticancer potential of GC, a dimeric stilbenoid (resveratrol derivative). Nonmalignant cells were used to determine whether MSE or GC selectively targets cancer cells without affecting normal cells. Our findings indicate that MSE and GC inhibited the growth of human and mouse cancer cell types in a dose-dependent manner. Interestingly, GC significantly inhibited cell proliferation in human and murine cancer cells compared with *t*RV (*P* < 0.05), indicating that GC is more efficacious than *t*RV. Overall, our findings show that MSE and GC at clinically achievable concentrations inhibited cell proliferation in human and murine cancer cells significantly (*P* < 0.05), without affecting normal cells.

Accumulating data clearly indicate that induction of apoptosis is an important mechanism by which dietary compounds exhibit their chemopreventive potential 38–42. We investigated whether MSE or GC influences the cancer cells to undergo differentiation associated with apoptosis. Apoptosis induced by MSE or GC was confirmed by DAPI staining for specific morphological and nuclear material changes characteristic of apoptotic cells. Given that data from DAPI staining showed that MSE and GC induced apoptosis in all the cancer cells, MSE and GC likely inhibit tumor cell growth by inducing apoptosis (*P* < 0.001). In contrast to the profound apoptosis induction in cancer cells, only marginal or very low levels of apoptosis were detected in normal cells treated with MSE or GC.

Apoptosis, a programmed form of cell death, is a highly regulated biochemical process that occurs through a variety of distinct mechanisms. Members of the caspase family of cysteine proteases play a pivotal role in the effector phase of extrinsic and intrinsic apoptotic pathways 35,36. In an effort to better understand the possible mechanisms by which MSE or GC may regulate programmed cell death or apoptosis in cancer cells, we measured caspase-3/7 and -9 activities that are associated with the extrinsic and intrinsic apoptotic pathways, respectively. Our results provided evidence that MSE might induce apoptosis via caspase-3/7-independent mechanisms in poorly metastatic colon cells (HT-29), and MSE likely induce late stage apoptosis, in part by activating caspase-3/7 (extrinsic pathway), after prolonged (72 h) exposure of metastatic prostate cancer cells to MSE. On the other hand, our results provide evidence that GC might induce apoptosis in both HT-29 and PC-3 cells, at least in part via caspase-3/7 (extrinsic pathway)-dependent mechanisms. Thus, our findings provide evidence that GC might trigger both early and late stage apoptosis in cancer cells via extrinsic apoptotic pathway, at least in part by activating caspase 3/7-dependent mechanisms. However, caspase-9 (intrinsic pathway), an upstream protease of caspase-3/7 was not activated by MSE or GC in either HT-29 or PC-3 cells, suggesting that a proteolytic cascade involving activation of all caspases may not be a common or essential feature of MSE or GC-induced apoptosis. Apoptosis inducing factor (AIF) and endonuclease G are involved in initiating a caspase-independent pathway of apoptosis (positive intrinsic regulator of apoptosis) by causing DNA fragmentation and chromatin condensation. Future studies will be designed to investigate the roles of AIF and endonuclease G in a caspase-independent apoptosis pathway, to determine the exact molecular mechanisms associated with the proapoptotic effects of MSE and GC. Thus, for the first time, our study demonstrated that both MSE and GC could be potential candidates for novel cancer chemopreventive agents.

Validation of the in vitro antitumor activity of an agent in an in vivo setting is important to determine its potential use as a chemotherapeutic compound to prevent and treat cancer. Colon cancer is one of the most common prevalent cancers among males and females 43,44. Therefore, in the present work, the antitumor activity of MSE observed at the in vitro was validated in a preclinical setting in vivo. Animal models are extremely critical to assess the efficacy of antitumor agents in the preclinical settings. The subcutaneous colon tumor models are still used to evaluate treatment efficacy against colon tumor growth 45,46, including tumor metastasis to the liver 45. The colon-26 mouse model, whereby the murine colon-26 cells are injected subcutaneously into the flank of mice, is commonly used to study the effects of several antitumor agents, including dietary agents 47–51. The colon-26 cells are highly tumorigenic and have the tendency to metastasize. Therefore, female BALB/c mice implanted with colon-26 cells were used to evaluate the antitumor efficacy of MSE. Our results show that a low dose of MSE (50 mg/kg) significantly suppressed colon tumor growth in BALB/c mice bearing colon-26 tumors, and inhibited tumor-induced intratumoral angiogenesis effectively than a high dose of MSE (100 mg/kg). However, there were no significant differences in the tumor volume and tumor weight between the 50 and 100 mg/kg doses. Interestingly, our results are consistent with earlier reports that natural products at lower concentrations exert more antitumor effectives than high concentrations 52–55. The precise reason why the MSE or other natural products did not show a dose-dependent antitumor efficacy is unknown. A possible explanation is that accumulation of nonprotective MSE constituents restricted the beneficial effects of high-dose MSE.

Liver metastases are frequently inoperative and significantly affect the prognoses of patients with cancer 37. The subcutaneous colon tumor models do not robustly form metastases in the liver. Therefore, to evaluate the effect of MSE on metastasis, colon-26 tumor cells were implanted into the spleens of female BALB/c mice to produce liver metastasis (tumor nodules), as reported previously 47–51,56. Our findings also revealed that MSE inhibits the formation of metastatic nodules on the liver surface and reduces liver metastasis in these mice. However, there were no significant differences in the antitumor or antimetastatic activities between the lower and higher doses of MSE treatment.

In conclusion, our findings provide the first evidence that MSE and its active ingredient GC, both of which are known to have excellent bioavailability and safety, also have potent antitumor activity. This suggests that both MSE and GC might be effective for the prevention and treatment of certain cancers. Most importantly, our observations, for the first time, suggest that MSE inhibits tumor growth, intratumoral angiogenesis, and liver metastasis in a colon-26 tumor-bearing model. A humanized orthotopic colon tumor model would provide valuable insights into the effects of MSE on preventing tumor growth and spontaneous metastases from the colon to the liver and/or lung. However, this is beyond the scope of the current efforts which seeks mainly to determine the antitumor potential of MSE using both the in vitro and in vivo tumor models to better assess its clinical potential to prevent and/or treat human colon cancer. To this end, we will conduct further preclinical studies that use a humanized orthotopic colon tumor model that involves the orthotopic transplantation of xenograft tumors in the primary colon to generate spontaneous liver and/or lung metastases.

## Supporting Information

**Figure S1.** Effects of MSE and gnetin C on caspase-3/7 activity in HT-29 and PC-3 cells. HT-29 and PC-3 cells were treated with MSE (25 and 50 µg/mL) or gnetin C (6.3 and 12.5 µM) around the IC_50_ concentration of each cell line (determined by a dose-response study for 48 h) for 6, 24, 48, or 72 h. The effects of MSE and gnetin C on the activity of caspase-3/7 were determined at each time point. The bar graph shows the percentage of caspase-3/7 activity normalized data with vehicle (control) at each time point. The data represent the mean ± SD (*n* =3). **p*<0.05, compared to control at each time point.

**Figure S2.** Effects of MSE and gnetin C on caspase-9 activity in HT-29 and PC-3 cells. HT-29 and PC-3 cells were treated with MSE (25 and 50 µg/mL) or gnetin C (6.3 and 12.5 µM) around the IC_50_ concentration of each cell line (determined by a dose-response study for 48 h) for 6, 48, or 72 h. The effects of MSE and gnetin C on the activity of caspase-9 were determined at each time point. The bar graph shows the percentage of caspase-9 activity normalized to the vehicle (control) at each time point. The data represent the mean ± SD (*n* = 3). No significant activation of caspase-9 was detected with MSE or GC treatment.
