# Supplementary figures and images for: Antitumor activity of melinjo (Gnetum gnemon L.) seed extract in human and murine tumor models in vitro and in a colon-26 tumor-bearing mouse model in vivo
Source: Cancer Med. 2015 Sep 26;4(11):1767–80. doi: 10.1002/cam4.520 (PMC4674003; doi:10.1002/cam4.520)

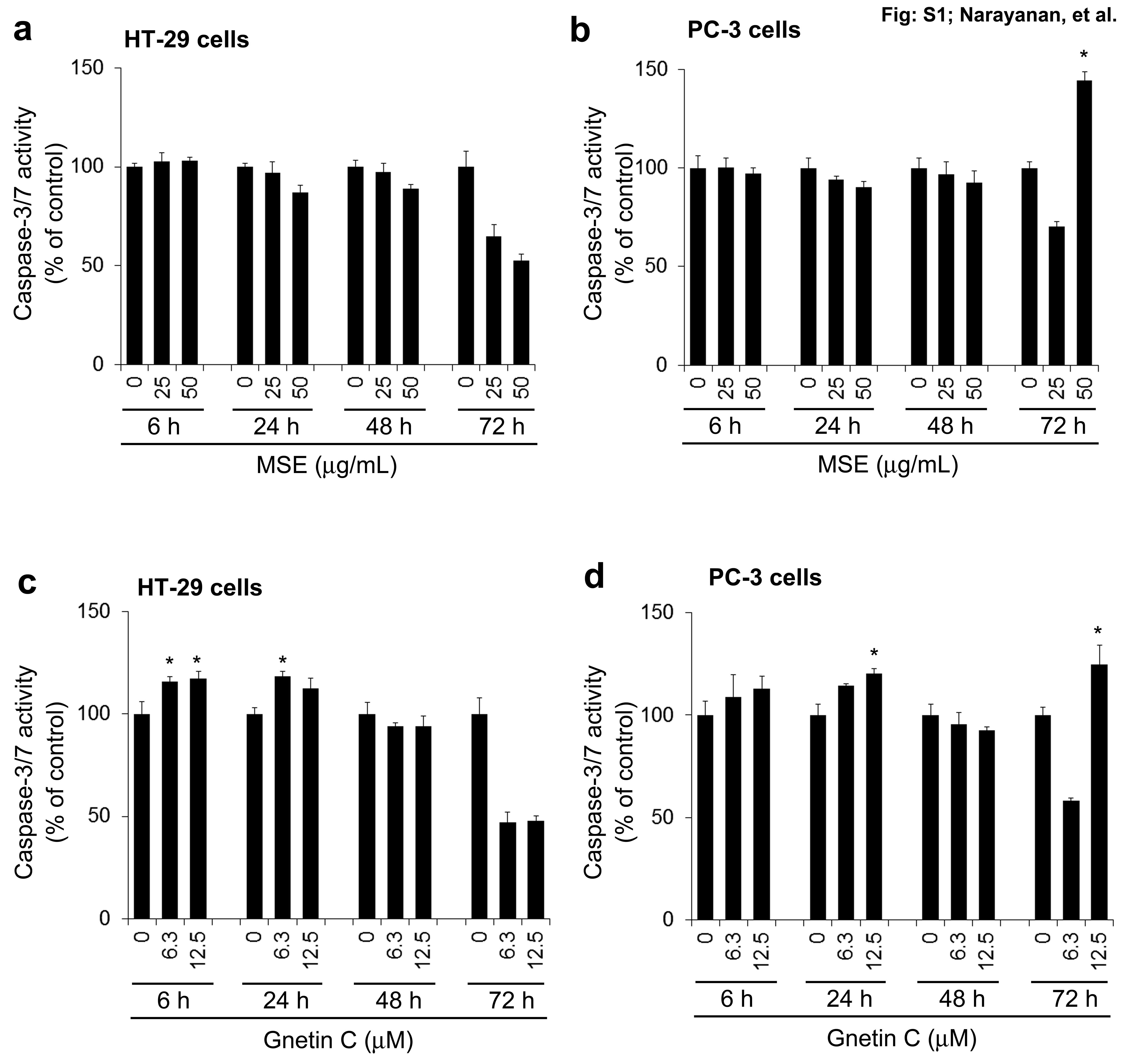

Supplement: Supplementary file 1 [file cam40004-1767-sd1.jpg]

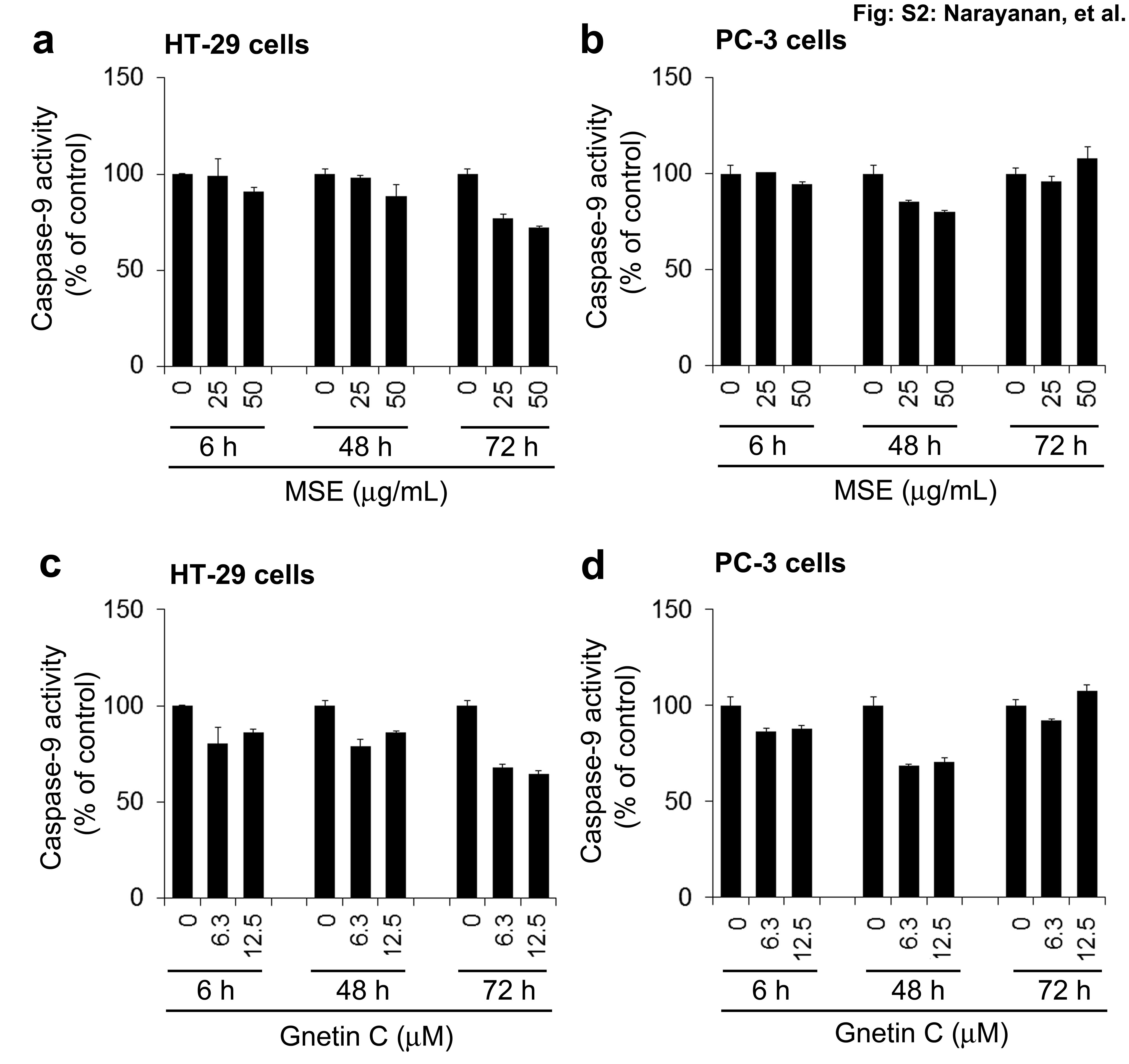

Supplement: Supplementary file 2 [file cam40004-1767-sd2.jpg]
